# Activatable fluorescence detection of epidermal growth factor receptor positive mediastinal lymph nodes in murine lung cancer model

**DOI:** 10.1371/journal.pone.0198224

**Published:** 2018-06-01

**Authors:** Xieyi Zhang, Takahito Nakajima, Mai Kim, Aiko Yamaguchi, Oyunbold Lamid-Ochir, Huong Nguyen-Thu, Anu Bhattarai, Hirofumi Hanaoka, Yoshito Tsushima

**Affiliations:** 1 Department of Diagnostic Radiology and Nuclear Medicine, Gunma University Graduate School of Medicine, Maebashi, Japan; 2 Department of Bioimaging Information Analysis, Gunma University Graduate School of Medicine, Maebashi, Japan; 3 Research Program for Diagnostic and Molecular Imaging Division of Integrated Oncology Research, Gunma University Initiative for Advanced Research, Maebashi, Japan; University of South Alabama Mitchell Cancer Institute, UNITED STATES

## Abstract

It is important to detect mediastinal lymph node metastases in patients with lung cancer to improve outcomes, and it is possible that activatable fluorescence imaging with indocyanine green (ICG) can help visualize metastatic lymph nodes. Therefore, we investigated the feasibility of applying this method to mediastinal lymph node metastases in an epidermal growth factor receptor (EGFR)-positive squamous cell carcinoma of the lung. Tumors were formed by injecting H226 (EGFR-positive) and H520 (EGFR-negative) cell lines directly in the lung parenchyma of five mice each. When computed tomography revealed tumors exceeding 8 mm at their longest or atelectasis that occupied more than half of lateral lung fields, a panitumumab (Pan)–ICG conjugate was injected in the tail vein (50 μg/100 μL). The mice were then sacrificed 48 hours after injection and their chests were opened for fluorescent imaging acquisition. Lymph node metastases with the five highest fluorescent signal intensities per mouse were chosen for statistical analysis of the average signal ratios against the liver. Regarding the quenching capacity, the Pan–ICG conjugate had almost no fluorescence in phosphate-buffered saline, but there was an approximate 61.8-fold increase in vitro after treatment with 1% sodium dodecyl sulfate. Both the fluorescent microscopy and the flow cytometry showed specific binding between the conjugate and H226, but almost no specific binding with H520. The EGFR-positive mediastinal lymph node metastases showed significantly higher average fluorescence signal ratios than the EGFR-negative ones (n = 25 per group) 48 hours after conjugate administration (70.1% ± 4.5% vs. 13.3% ± 1.8%; *p* < 0.05). Thus, activatable fluorescence imaging using the Pan–ICG conjugate detected EGFR-positive mediastinal lymph node metastases with high specificity.

## Introduction

Lung cancer is one of the most frequently diagnosed cancers and leading causes of cancer-related death worldwide [1]. Moreover, it remains the primary cause of death from malignancy in the United States of America [2]. In addition to standard therapeutic strategies of lung cancers including surgery, chemotherapy, and radiotherapy, the molecular therapy targeting the epidermal growth factor receptor (EGFR) transmembrane glycoprotein has been developed in recent decades [3–6]. The EGFR status has mainly been evaluated by fluorescence in situ hybridization or immunohistochemistry [7], both of which require invasive procedures. The imaging modalities may also offer a more comprehensive means of evaluating EGFR status [8, 9]. Since FDG-PET can visualize the glucose metabolism, primary cancers, metastatic lesions and lymph node metastases can be detected [10, 11]. However, the residual lesions or lymph node metastases can be difficult to detect during surgery for lung cancer, which increases the risk of untreated cancer and leads to a poorer prognosis [12]. Intra-operative fluorescence-guided methods is emerging as a viable technique for the complete resection of cancer [8].

In vivo fluorescence imaging is a new modality that has been used to provide more information about cancer during surgery [13]. Near-infrared fluorescence imaging with indocyanine green (ICG) has an advantage in improving tissue penetration and ICG also has a long history of being used clinically [14]. Fluorescence imaging with near-infrared light enables lesions to be visualized even in deep tissue, including in mediastinal lymph node metastases [15]. One challenge in fluorescence imaging is to activate the fluorescence ability of probes using activatable method. Kobayashi, et. al has reported an activatable method that enables lesions to be brightened with low background fluorescence signals [16–18]. The activatable method allows the fluorescence imaging probes to switch their status from the quenched (off) to the active state (on) and it improved the target-to-background ratios. The process works by only showing metastatic lymph nodes as bright lesions, leaving nodes with quenched fluorescence dye to produce no signals.

In the current study, we investigated whether lymph node metastases of lung cancer can be detected using the activatable method, by examining EGFR-positive lung squamous cell carcinoma in murine models. This could be a first step towards enabling lesion detection by intra-operative imaging. Since video-assisted thoracic surgery (VATS) is a widely available procedure that utilizes a charge coupled device camera to view images with not only visible light but also near-infrared light [19], this activatable method could eliminate the need for rapid pathological diagnosis during surgery of lung cancer [20].

## Materials and methods

### Synthesis of fluorophore conjugated antibody

The synthesis of fluorophore conjugated antibody was carried out as reported previously [16]. A humanized monoclonal antibody, panitumumab (Pan) (1 mg, 6.8 nmol), was incubated with ICG-Sulfo-OSu (66.8 μg, 34.2 nmol, 5 mmol l^−1^ in dimethyl sulfoxide, Dojindo, Inc., Japan) in 0.1 M Na_2_HPO_4_ (pH 8.5) at room temperature for 30 min. The reaction molecule ratio of Pan and ICG-Sulfo-OSu was 1 to 8. The mixture was then purified with a Sephadex G50 column (PD-10; GE Healthcare, Piscataway, NJ). The protein concentration was determined and the number of fluorophore molecules conjugated to each antibody molecule (i.e., Pan-ICG conjugates) was confirmed as 1 to 1.93 with a UV-vis system (model 8453UV-visible value system; Agilent Technologies, Santa Clara, CA).

### Validation of quenching ability in vitro

For the validation of the quenching and dequenching ability of bound ICG, the Pan-ICG conjugates were treated with 1% sodium dodecyl sulfate to diminish hydrophobic k–k interactions and separate immunoglobulin G chains. The change in fluorescence intensity of the conjugate was measured by a Maestro In Vivo Imaging System (CRI Inc., Woburn, MA), using laser excitation at 785 nm and emission at 820 nm.

### Cell culture

EGFR-positive and EGFR-negative human squamous cell carcinoma cell lines were employed for the EGFR targeting studies (H226 and H520, respectively) [21]. Both cells were seeded in Roswell Park Memorial Institute-1640 medium (Life Technologies, Gaithersburg, MD) supplemented with 10% fetal bovine serum (Life Technologies, Gaithersburg, MD) and 1% penicillin (Biofluids, Camarillo, CA). These were placed in a humidified incubator at 37°C in an atmosphere of 95% air and 5% carbon dioxide.

### Flow cytometry

H226 or H520 cells (1 × 10^5^ cell/well) with Pan-ICG were incubated at 37°C for either 1 or 6 hours. To validate the specific binding of the antibody, excess Pan (50 μg) was used to block 0.5 μg of Pan-ICG conjugate. The fluorescence signal from H226 and H520 cells after incubation with the Pan-ICG conjugate was measured by flow cytometry (BD Biosciences, San Jose, CA).

### In vitro fluorescence microscopy studies

The H226 or H520 cells (1 × 10^4^) were plated on a covered glass-bottomed culture well and were incubated for 24 hr. Next, the Pan-ICG was added to the medium (5 μg/mL) and the cells were incubated for either 1 or 6 hr. The cells were then washed once with phosphate-buffered saline, and were subjected to fluorescence microscopy using a BZ-X700 microscope (Keyence, Osaka, Japan) equipped with filters meeting the following criteria: excitation wavelength 672.5–747.5 nm and emission wavelength 765–855 nm. Transmitted light differential interference contrast images were also acquired.

### Animal tumor model

All the procedures were carried out in compliance with the Guide for the Care and Use of Laboratory Animal Resources (1996), National Research Council, and approved by the local Animal Care and Use Committee. Ten female, athymic nu/nu mice (Japan SLC, Inc., Shizuoka, Japan) were used for all the experiments from age 4 weeks (weight, 9–11 g). All the mice were raised at room temperature (25 ± 2) °C and given aseptic full-price nutritional pellet feed and sterile water. The mice were randomly segregated into two groups of five mice each, and H226 and H520 cells suspended in phosphate-buffered saline (1–3 × 10^6^ cells/50 ml) were injected into the lung tissue directly in each group, respectively. A 24G needle was inserted perpendicular to the chest wall at the midpoint between the diaphragm and axilla on the right or the left axillary line at a 4mm depth for this injection. After the injection of the tumor cells, the needle was promptly pulled out to avoid pneumothorax and additional damage. The mice were observed until complete recovery from anesthesia. During the whole procedure of injection, the mice were anesthetized with 5% of isoflurane (Wako Pure Chemical Industries, Osaka, Japan). The number of mice selected was determined based on the Power and Sample Size Calculation program developed by Dupont and Plummer [22].

### Tumor monitored by computed tomography

After injecting the tumor cells, tumor growth was monitored by animal computed tomography (CT; LaTheta LCT-200; Hitachi-Aloka, Tokyo, Japan) every week. During the procedure of CT monitoring, the mice were anesthetized with 5% of isoflurane (Wako Pure Chemical Industries, Osaka, Japan) and fixed in a plastic bed. Only the images within the lung were acquired to reduce the duration of the experiment. Animal models were daily observed and were thought to be established for this in vivo study when the longest diameter of the lung tumor lesion exceeded 8 mm or atelectasis occupied more than half of the lateral lung fields.

### In vivo EGFR-targeted imaging studies

The Pan-ICG conjugate was diluted in 100 μL of phosphate-buffered saline, and a dose of 50 μg was injected via the tail vein into the tumor-bearing mice. Fluorescence images of opened chest and ex vivo images of LN and liver were obtained using the Maestro In Vivo Imaging System. Forty-eight hours after conjugate administration, all the mice were sacrificed by cervical dislocation under deep anesthesia with 5% of isoflurane (Wako Pure Chemical Industries, Osaka, Japan) air. After their chests were opened, their lungs were collapsed. Then, their hearts were removed for fluorescent imaging. The regions of interest were drawn for the five lymph node metastases with the highest fluorescent signal intensities per mouse, and the fluorescence signal intensity was calculated. The ratio of the fluorescence signal intensity between lymph node metastases and the liver was used for statistical comparison. The lung tumor size was calculated by following formula after excision: size = [length × width^2^] / 2. The correlation between the lesion size and the signal ratio was analyzed.

### Ex vivo EGFR-targeted imaging studies

To confirm the presence of lymph node metastases and EGFR expression, lymph nodes were embedded in paraffin and stained with hematoxylin and eosin and the Pan-ICG conjugate. Fluorescence microscopy and optical images (hematoxylin and eosin staining) were acquired.

### Statistical analysis

All the data are expressed as means ± standard deviations. Quantitative data were analyzed by independent-sample Student *t*-tests, and the correlation between tumor size and fluorescence signal intensity was analyzed by Pearson’s correlation. IBM SPSS version 24.0 (IBM Corp., Armonk, NY) was used for all data analyses and significance was set at *p* < 0.05.

## Results

### Validation of quenching ability in vitro

**Fig 1:**
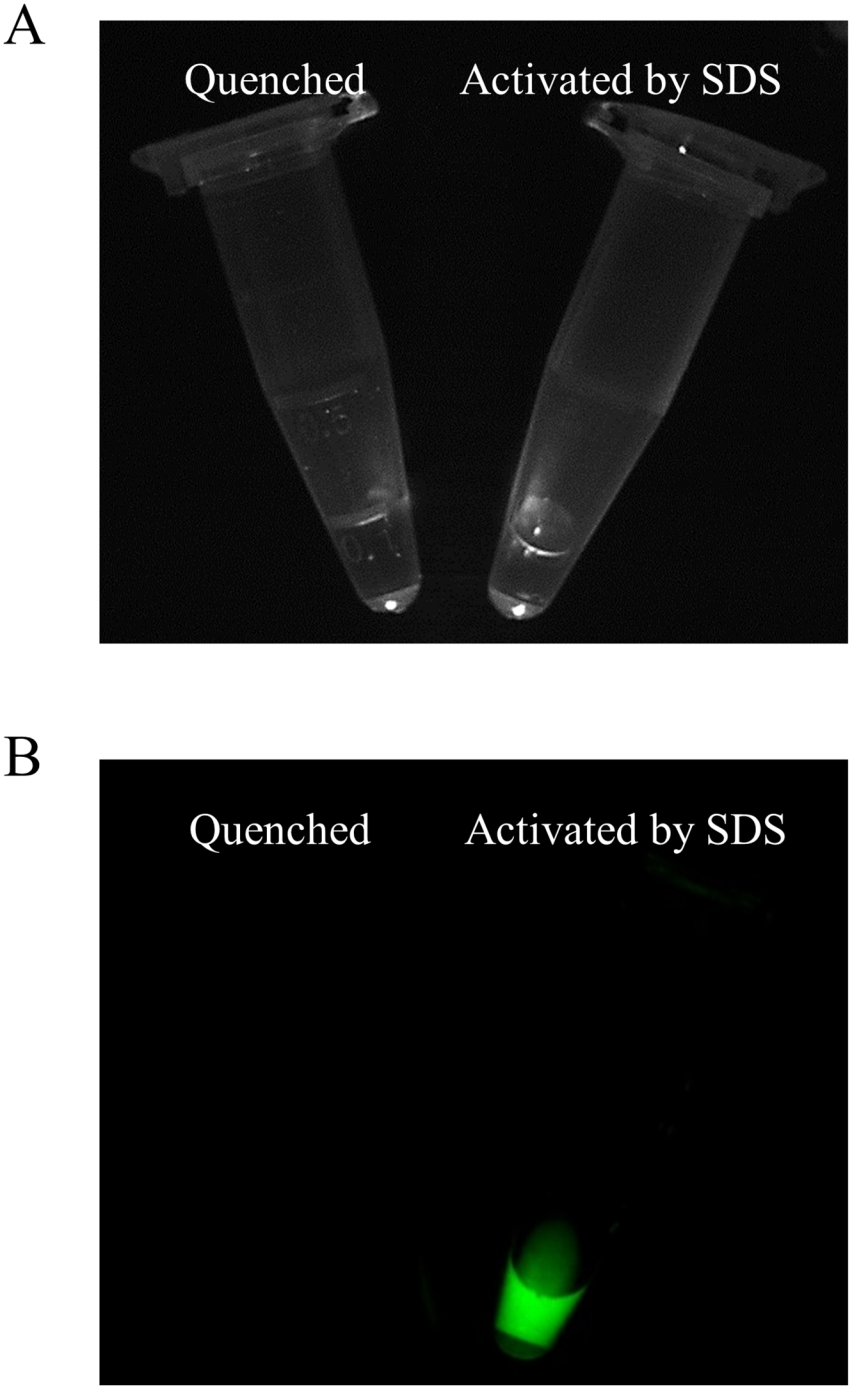
Quenched (left) and chemically dequenched (right) Pan-ICG. (A) White image (B) Fluorescence image. Dequenched conjugates showed increased fluorescence signals as shown in green.

After exposure to 1% sodium dodecyl sulfate, a 61.8-fold increase in fluorescence signals was detected for the Pan-ICG (Fig 1).

### Flow cytometry

**Fig 2:**
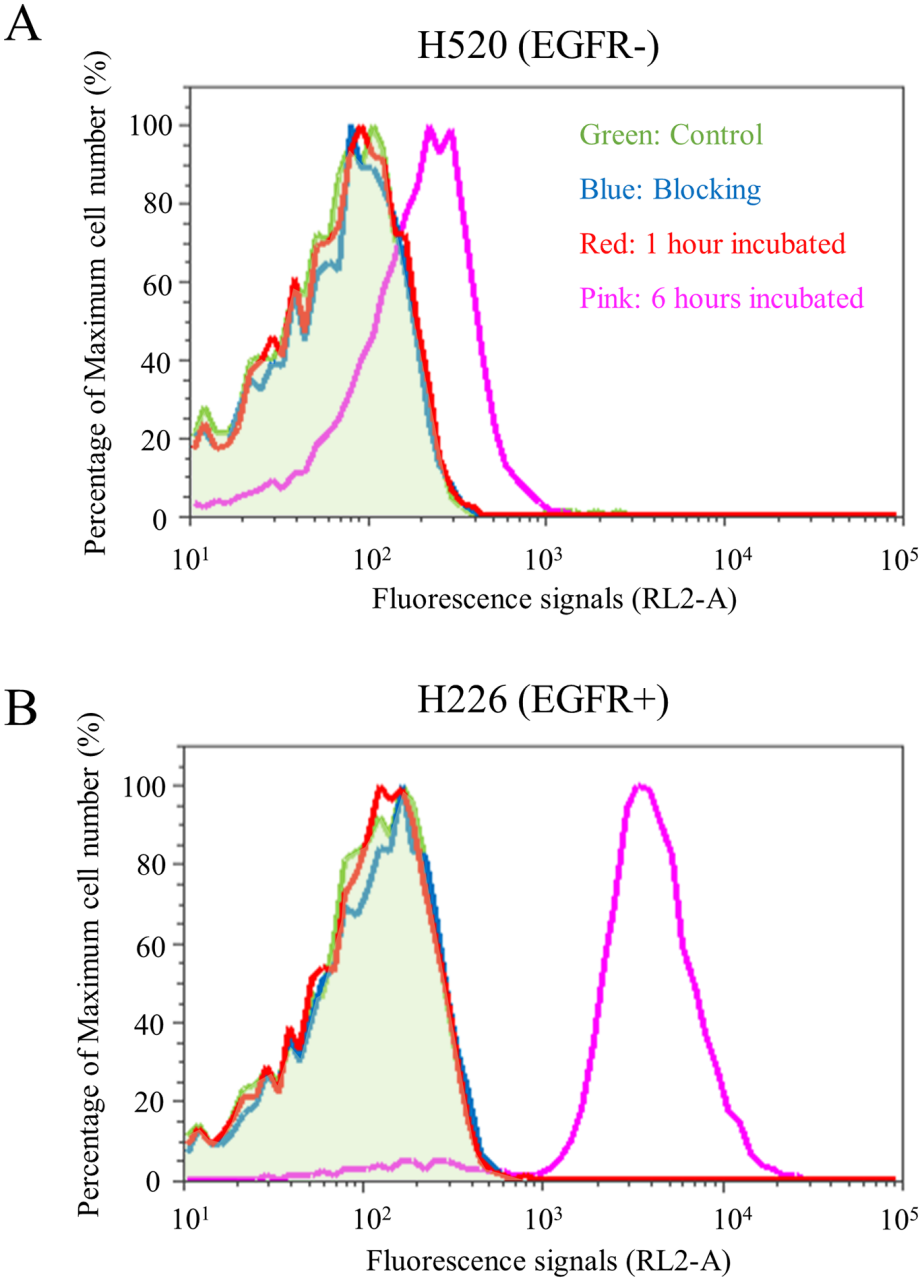
Flow cytometry analysis of H520 and H226 cell lines binding to Pan-ICG. In vitro cell experiment with flow cytometry showed (A) non-specific weak binding of Pan-ICG to EGFR- cell line at 6 hours and (B) specific binding of Pan-ICG to EGFR+ cell line at 6 hours.

Flow cytometry using the Pan-ICG showed strong fluorescence from the EGFR-positive cells and weak fluorescence from the EGFR-negative cells after incubation with the Pan-ICG for 1 or 6 hours. Given that this weak binding could not be blocked with a 100-fold excess of unlabeled Pan antibody, it was considered to represent low-level, nonspecific activation of the quenched ICG, which was only seen in vitro (Fig 2).

### In vitro fluorescence microscopy

**Fig 3:**
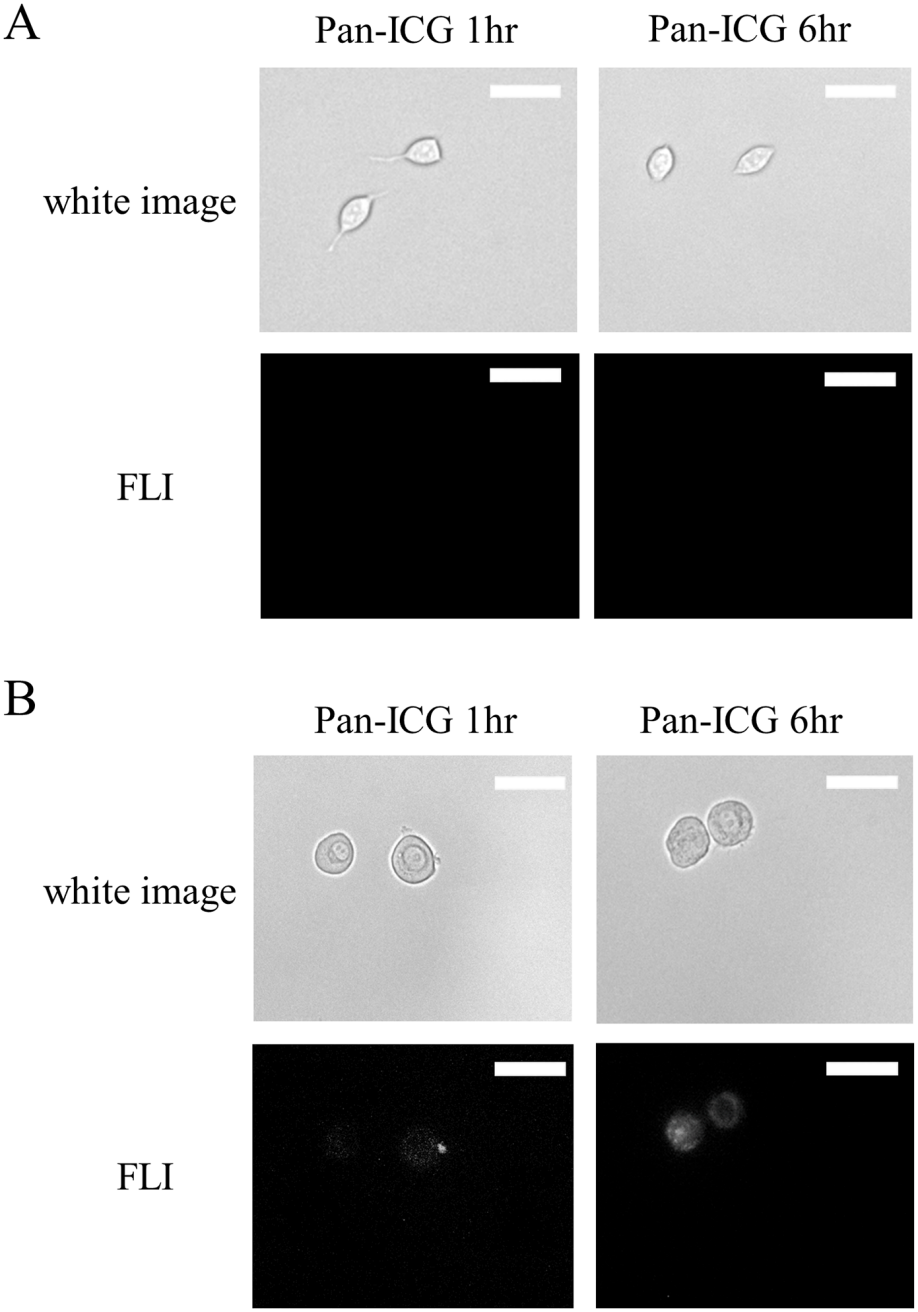
Fluorescence microscopy study of Pan-ICG binding to H520 and H226 cell lines. After 1 hour (1hr) and 6 hours (6hr) incubation, fluorescence microscopy results showed (A) no signal from EGFR- cell line (H520) and (B) weak signals at 1hr or strong signals at 6hr based on the specific binding of conjugate to EGFR+ cancer cell line (H226). Scale bar indicates 20 μm.

In vitro fluorescence microscopy produced similar results to those obtained for flow cytometry. Images after 1 and 6 hours’ incubation showed increased signal intensity in the EGFR-positive tumor cells (Fig 3).

### Tumor monitored by CT

**Fig 4:**
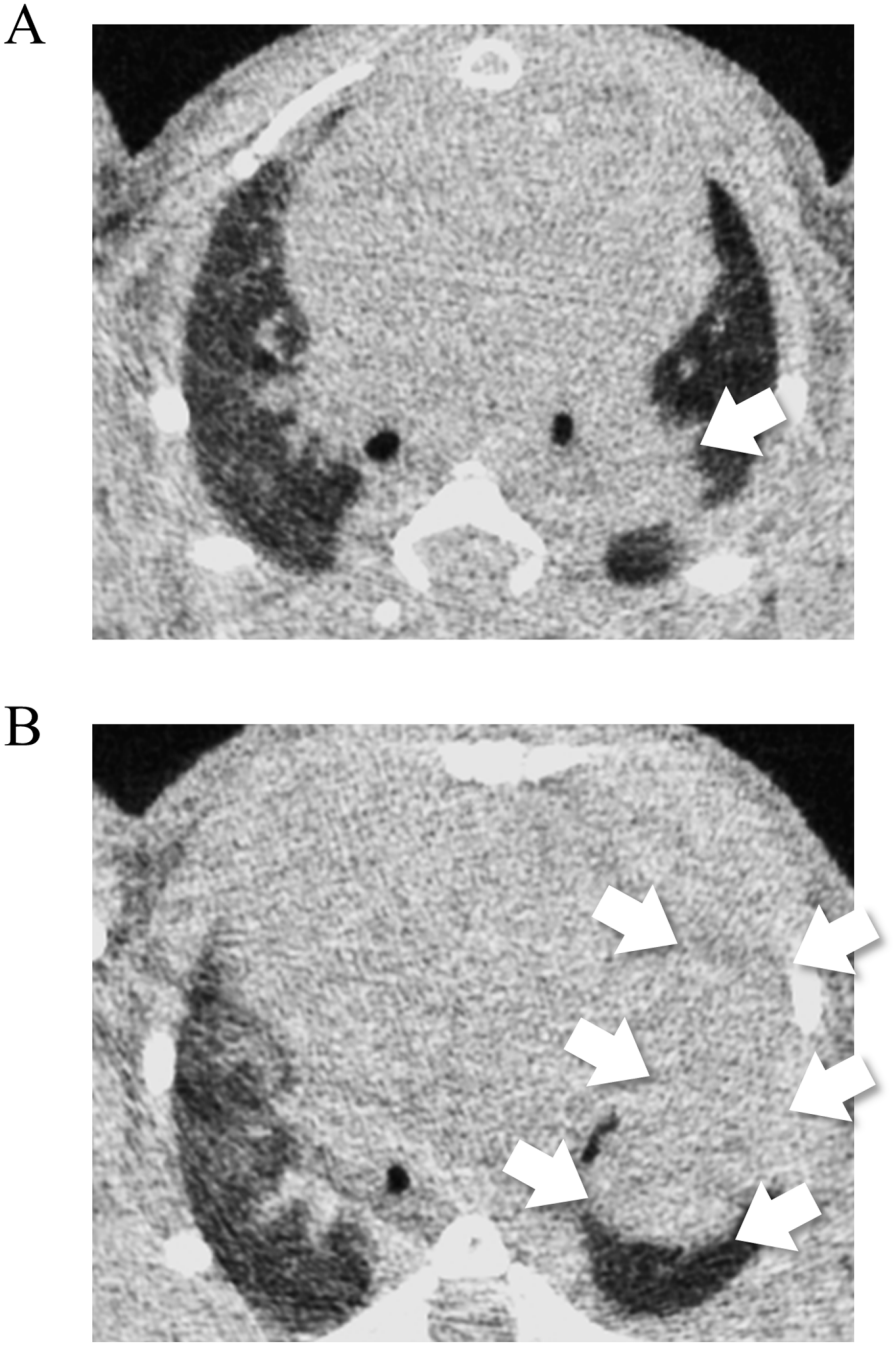
Tumor monitored by animal CT. (A) The CT image 63 days after tumor cell implantation. The longest diameter of the tumor lesion was 8.3 mm in the left lung. (B) The CT image 56 days after tumor cell implantation. The left lung showed more than half atelectasis.

The imaging experiments were performed 56 to 98 days (74.9 ± 13.6 days) after cell injection, when the CT criteria were met (Fig 4).

### In vivo EGFR-targeted imaging studies

**Fig 5:**
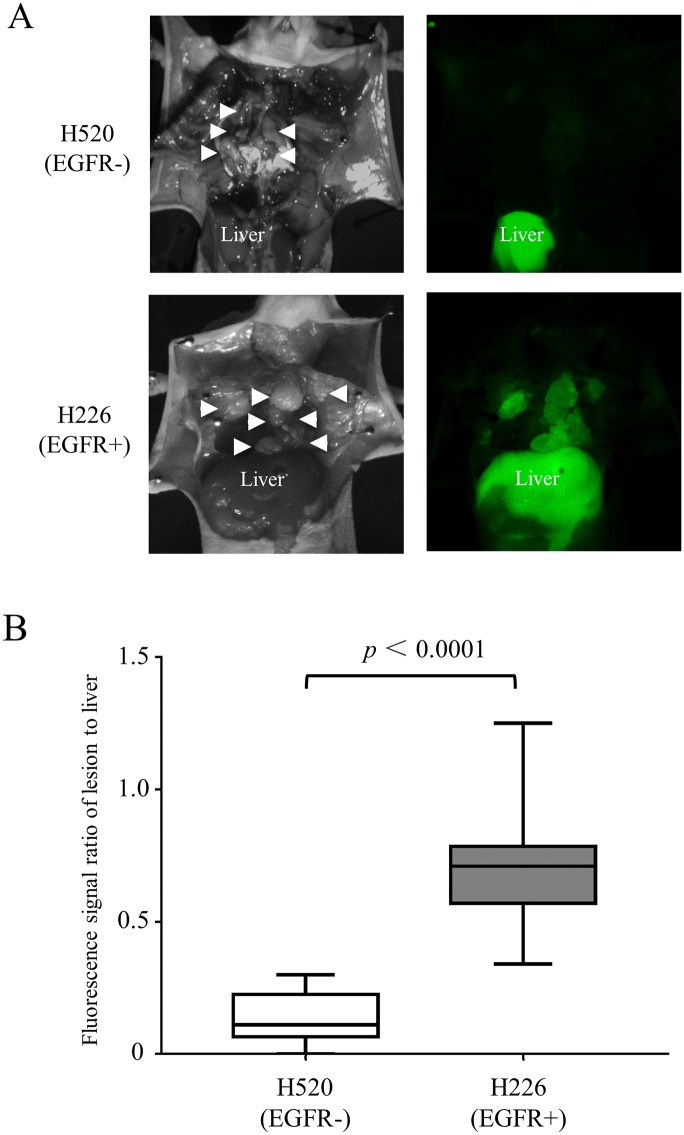
In vivo EGFR targeted imaging. (A) In vivo fluorescence images of an EGFR- (upper) and EGFR+ (lower) tumor bearing mouse injected with 50 μg of Pan-ICG. EGFR+ lymph node metastases (white arrow heads) showed high fluorescence signals while no signals from EGFR- ones were seen. On both images, the liver showed high signals with non-specific accumulations. (B) Comparison of the fluorescence signal ratio of lesion to liver between EGFR+ and—lymph node metastases 48 hours after injection of 50 μg Pan-ICG. The signal ratio was significantly higher in EGFR+ lymph node metastases than in EGFR- ones (*p*< 0.05).

**Fig 6:**
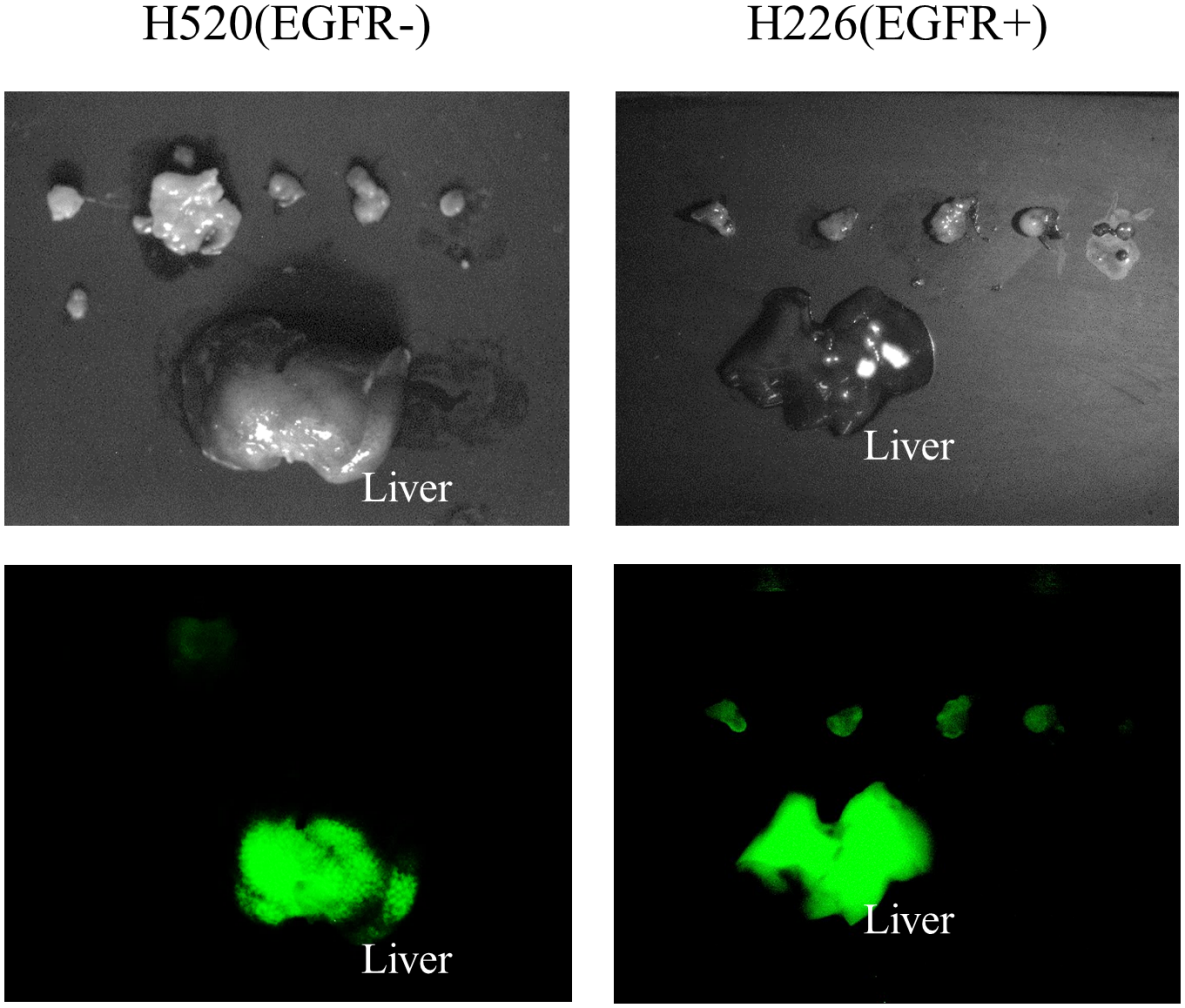
Ex vivo fluorescence images of EGFR- and EGFR+ lymph node metastases. Ex vivo fluorescence images showed higher fluorescence signals in EGFR+ lymph node metastases than in EGFR- ones.

**Fig 7:**
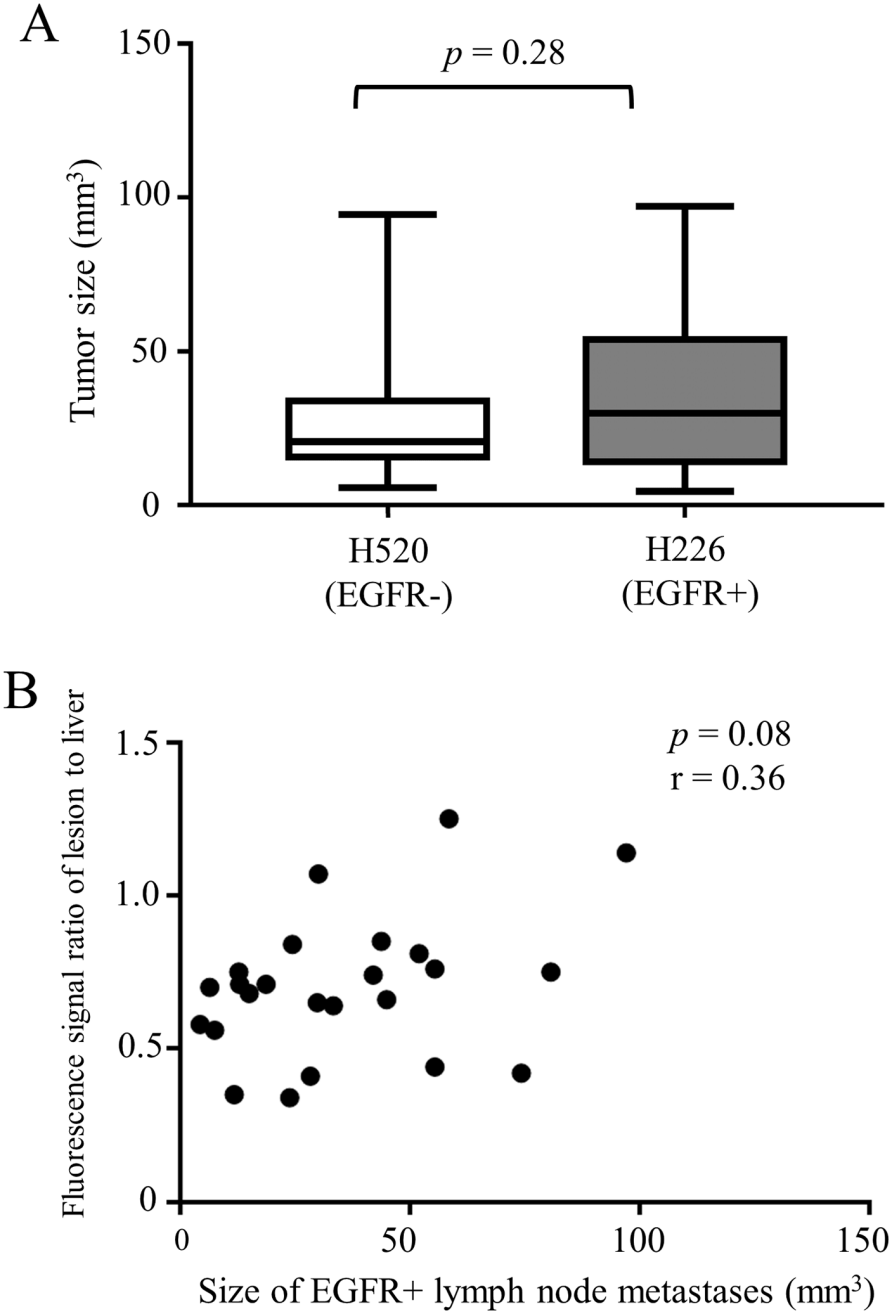
Comparison of the tumor size between EGFR- and EGFR+ lymph node metastases. (A) There was no significant difference in tumor size between EGFR- and EGFR+ metastases. (B) Correlation between the fluorescence signal ratio of the lymph node metastases to liver and the size of EGFR+ lesions.

The EGFR-positive node metastases had significantly stronger fluorescent signal intensities than the EGFR-negative nodes 48 hours after injection. All the 25 EGFR-positive lymph node metastases were clearly identified with the ICG-specific fluorescence signal, while the EGFR-negative tumor specimens produced a much weaker signal. Given that fluorescence from scattered EGFR-negative metastases was practically equivalent to the background level, signals from them were eliminated from calculations. The lesion-to-liver fluorescence signal ratio was calculated (Fig 5), with a significantly higher signal ratio in the EGFR-positive lymph node metastases (*p* < 0.05). Visually, ex vivo images showed the EGFR-positive lymph node metastases acquired higher fluorescent signals than the EGFR-negative ones (Fig 6). The tumor size was not significantly different between the EGFR-positive (35.4 ± 24.7 mm^3^; range, 4.5–80.8 mm^3^) and the EGFR-negative (28.2 ± 22.3 mm^3^; range, 5.7–94.4 mm^3^) lymph node metastases (*p* = 0.28), and the signal ratio did not correlate with the lesion size in the EGFR-positive lesions (Fig 7, r = 0.36, *p* = 0.08).

### Ex vivo EGFR-targeted imaging studies

**Fig 8:**
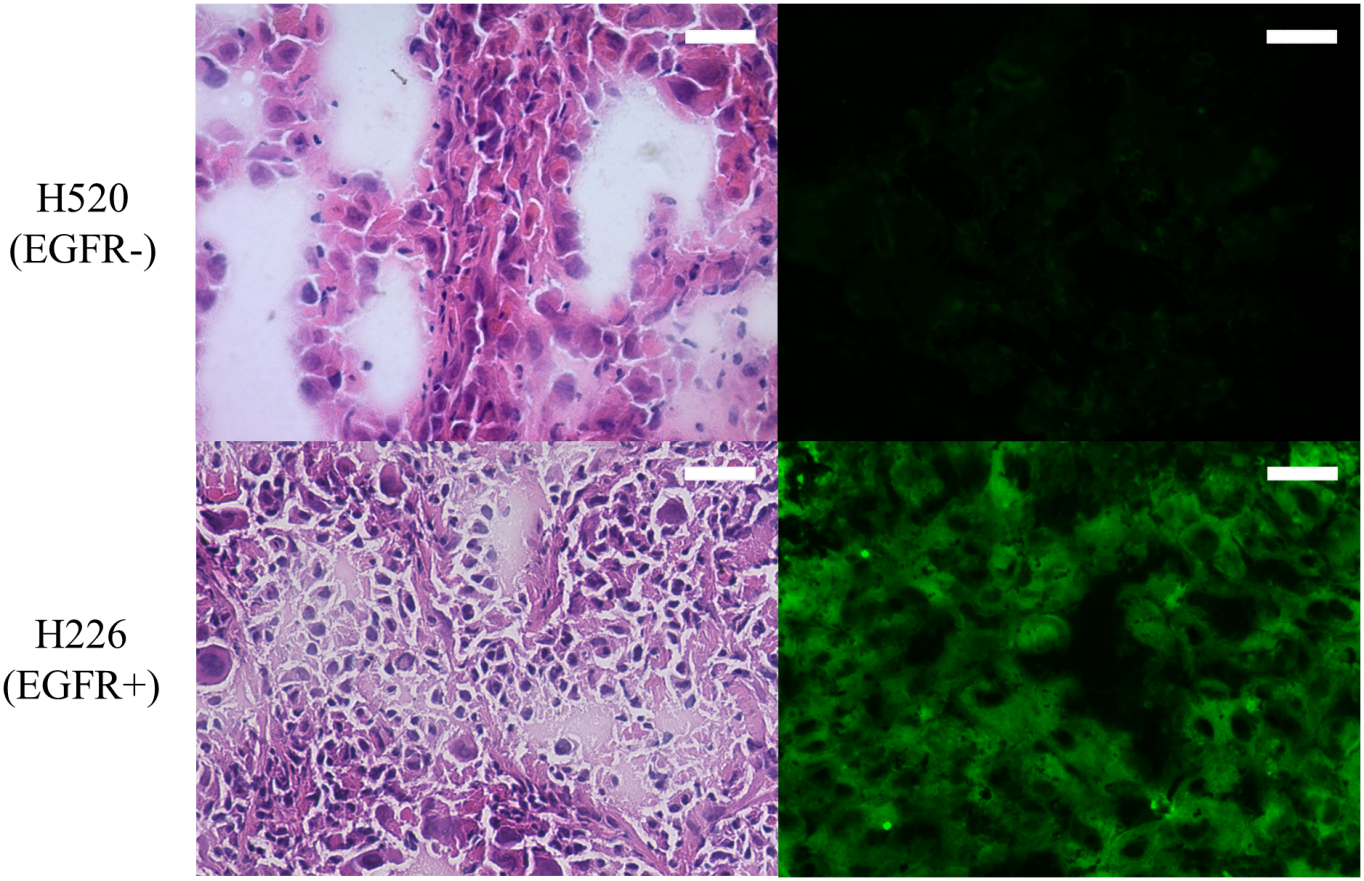
Pathological and immunohistochemical examinations. Comparison of the HE staining (Left) and Pan-ICG staining (Right) between EGFR- and EGFR+ lymph node metastases. Scale bar indicates 20 μm.

We confirmed the presence of lymph node metastases in all 50 lesions by staining with hematoxylin and eosin. At 30 minutes after incubation with Pan-ICG, the EGFR-positive tumors showed stronger fluorescent signal intensity than the EGFR-negative tumors (Fig 8).

## Discussion

ICG itself is widely applied in clinical settings such as ophthalmic or intraoperative angiography and lymphangiography for sentinel lymph node detection where ICG binds to serum albumin, resulting in an enhancement of its fluorescence ability. Since the half-life of ICG in human body is about 3 to 4 minutes and ICG is metabolized by the liver, ICG is also clinically used as an index for liver function.

In the present study, ICG was employed as an activatable fluorescence dye. Since molecules of ICG were abundantly conjugated to an antibody, ICG loses its fluorescence by the fluorescence resonance energy transfer (FRET). ICG has been studied as an activatable fluorescent dye and it has been shown that quenched ICG regains fluorescence ability after binding to target antigens, internalization of target cells and then metabolization in lysosomes [23]. Using a fluorescent near-infrared dye also allows deeper tissue penetration than can be achieved with visible light alone [14]. Studies have shown that ICG conjugated with a monoclonal antibody required 24 to 48 hours to be sufficiently taken up, internalized, and activated by tumor cells. However, the resulting signal can persist for at least 10 days after administration if activatable fluorescence imaging technique is employed [15, 16].

We showed that Pan-ICG is promising as an activatable probe for use in the detection of lymph node metastases in EGFR-positive squamous cell cancer of the lung. Flow cytometry and fluorescence microscopy studies have consistently shown that the quenched Pan-ICG conjugate can be activated only after binding to EGFR on the surface of cancer cells, and cannot activate EGFR-negative lymph node metastases. We found its ability to be activated in only EGFR-positive lesions led to very high target-to-background ratios. The minimum diameter of lymph node metastases among every 5 lymph nodes in each mouse was 2.1 mm. However, in vivo fluorescence imaging detected lymph nodes with a diameter of less than 2 mm. This finding indicates that micrometastatic lesions can be detected with this activatable method with near-infrared light. Even in inoperable cases, an activatable antibody could be useful because expression of membrane antigens can be confirmed with VATS following an administration of such antibody for prediction of the efficacy of therapy [24].

Activatable fluorescence imaging using ICG conjugated with an antibody has been widely studied. As mentioned in a previous murine study, activatable J591-ICG was shown to be a promising molecular imaging probe for detecting both primary and metastatic prostate cancer if positive for the prostate-specific membrane antigen [16]. In addition, daclizumab conjugated with ICG was shown to have high tumor-to-background fluorescence in CD25-expressing tumors. Furthermore, tumors overexpressing HER1 and HER2 have been successfully characterized in vivo based on Pan-ICG and trastuzumab-ICG [17].

In terms of the safety of using the Pan-ICG conjugate, both Pan and ICG have been approved by Food and Drug Administration (FDA) [25, 26]. Although the safety of Pan has been demonstrated in non-small cell lung cancer [27], the optimal dose still requires clarification because research has shown that conjugating ICG to Pan increased toxicity without a discernible impact on efficacy [28]. By contrast, ICG is an established clinical agent with more than 50 years’ experience, primarily in ophthalmology [25]. However, ICG-Sulfo-OSu is a chemically modified molecule of ICG and its safety has not yet been demonstrated. After the internalization of Pan-ICG, Pan would be metabolized and ICG-lysine might remain. Although ICG has a long history for clinical use, the toxicity of ICG-lysine or other metabolic forms should be investigated. Another issue in the clinical use of Pan-ICG system is an injection dose. The application of ICG for near-infrared imaging allows us to use ICG up to 5mg/ kg/ day. In this study, we injected Pan-ICG at the dose of 50 μg of Pan to a mouse intravenously. It matched 1.25 μg of ICG per a mouse. As a human dose, the total injection dose would be 2.5 mg per a human with 50 kg of weight. This amount was a dose that is 100 times lower than the daily maximum dose for a clinical use.

To apply a conjugate like Pan-ICG in the operating room, a near-infrared charge coupled device camera would be needed, as has been developed in recent years [29]: a new ICG imaging system, the HyperEye Medical System (Miziho Ikakogyo, Tokyo, Japan). The clinical efficacy and sensitivity of this to detect sentinel lymph nodes in patients with operable breast cancer was demonstrated in all the 168 patients included in the original research [30]. Furthermore, the HyperEye Medical System permitted the transcutaneous visualization of lymphatic vessels under light conditions, thereby facilitating the identification and detection of lymph nodes without affecting the surgical procedure [30].

There were some limitations in the present study. Our current finding on lymph node metastases applies only to EGFR-positive cells. Clinically, EGFR is overexpressed in 40 to 89 percent of non-small cell lung cancers [31]. However, that is not to say that activatable fluorescence imaging could never be used for EGFR-negative cells, if another target can be found for EGFR-negative cancer. Effort to find other surface markers of lung cancers is underway [32, 33]. We have previously reported the activatable probe targeting to HER2. The dye conjugate with trastuzumab, anti HER2 antibody, worked well as an activatable probe. HER2 antigens express on the surface of cell membrane in 6 to 35% of non-small cell carcinoma [34].

Since the diagnosis of primary lung cancers is achieved by biopsy before the operation in most cases, the information of surface antigens could be acquired from biopsy specimens through immunostaining. Once the target is determined from the pathological investigation of surface antigens, activatable fluorescence imaging is a promising method even for EGFR-negative cells since various antibodies can be used for making a conjugate with an antibody and ICG. Second, we investigated only one cell line with EGFR overexpression in the current study, but we would like to point out that the activatable fluorescence imaging could be used in any EGFR-positive cell lines since the accumulation of Pan-ICG is based on the “antigen–antibody” interaction. The number of surface antigen expressions would be different in each cell line. Since the mechanism of activatable fluorescence imaging could increase the signal-to-noise ratio when compared with the conventional fluorescence imaging, this method works in other cell lines with lower expression of EGFR.

## Conclusion

In conclusion, we have synthesized an activatable Pan-ICG conjugate and we have shown that this activatable conjugate can detect mediastinal EGFR-positive lymph node metastases of squamous cell carcinoma with such high contrast that this activatable Pan-ICG has a potential in assisting surgeons during endoscopic lung surgery.
